# Delayed heart rate recovery and its variability in fitness functional training compared to endurance athletes: a cross-sectional analysis

**DOI:** 10.7717/peerj.20335

**Published:** 2026-01-05

**Authors:** Freddy Enrique Ramos Guimarães, Michelle Teles Morlin, Kevin Alves Barreto, Luiz Guilherme Grossi Porto, Guilherme Eckhardt Molina

**Affiliations:** 1 Campus Morrinhos, Goiano Federal Institute, Morrinhos, Goiais, Brazil; 2 Faculdade de Educação Física, Programa de Pós-graduação em Educação Física, Laboratório de Fisiologia do Exercício, Universidade de Brasília, Brasília, DF, Brazil; 3 Grupo de Estudo em Fisiologia e Epidemiologia do Exercício e da Atividade Física (GEAFS), Universidade de Brasília, Brasília, DF, Brazil; 4 Rede SARAH de Hospitais de Reabilitação, Brasília, DF, Brazil

**Keywords:** Recovery, Exercise, Heart rate, Athletes, Cardiac autonomic function, Parasympathetic, Cardiac autonomic balance

## Abstract

**Introduction:**

The present study sought to expand upon prior investigations of the effect of different training modalities on post-exercise heart rate recovery (HRR) and heart rate variability (HRV).

**Objective:**

To compare the HRR and HRV in physically active men with endurance and fitness functional training recreational athletes following maximal treadmill exercise testing.

**Method:**

We conducted a cross-sectional study with 53 healthy men split into a control group (CG: *n* = 15; physically active men), endurance group (EG: *n* = 16; recreational triathlon athletes), and fitness functional training group (FFG: *n* = 15; recreational fitness functional training athletes). Short-term (5 min) HRR and HRV indexes (SD1, SD2, and HR/LF) were analyzed in 1-minute segments throughout 5 mins of recovery immediately following maximal treadmill exercise testing. Statistical analysis employed a generalized linear model test with a *p*-value set at 5%.

**Results:**

FFG showed slower HRR than EG at all post-exercise time points (β =  − 13.74 to −8.83 bpm; *p* < 0.01–0.02) and lower HRR than CG at the 1st minute of recovery (β =  − 10.47 bpm; *p* = 0.03). The SD1 (cardiovagal reactivation) was lower in CG than EG at the 1 st (β =  − 0.84 ms; *p* = 0.02); however, no differences (*p* > 0.05) existed among all groups throughout the recovery period. The SD2 (overall cardiac autonomic modulation) was lower in FFG than EG at 2nd (β =  − 2.51 ms, *p* = 0.02) and 3rd (β =  − 3.20 ms, *p* = 0.03) minutes. FFG and EG showed higher values of HR/LF index (indicative of sympathetic activity) than CG at the 1 st minute (β = 432.38 to 1,104.49; *p* < 0.01). FFG also showed higher HR/LF activity than CG an EG at the 2nd and 3rd minutes of recovery (β = 60.48 to 205.31, *p* < 0.01 to 0.05), and at the 4th minute, than EG (β = 29.33; *p* = 0.05).

**Conclusion:**

HRR was lower in FFG compared to EG throughout recovery. There were no differences in cardiovagal reactivation between the groups from the second to the fifth minute. FFG showed low cardiac overall modulation and high sympathetic activity throughout recovery compared to other groups. These findings may reflect that the FFG displays a persistent coactivation of both autonomic branches following maximal exercise testing compared to the different training modalities.

## Introduction

Post-exercise recovery refers to the period between the end of an exercise and the return to a resting or recovered state. It also encompasses peculiar physiological processes distinct from those of either the exercising or resting state. In this context, post-exercise heart rate (HR) recovery has been used as an independent marker of cardiovascular risk factors in healthy and diseased populations (Qiu et al., 2017; Stanley, Peake & Buchheit, 2013). Low heart rate recovery (HRR) is associated with higher cardiovascular morbimortality, especially in the first minutes of recovery (Cole et al., 1999). Conversely, faster HRR immediately following exercise is positively associated with improved cardiovascular function and athletic performance (Bellenger et al., 2016; Daanen et al., 2012).

The short-term HRR (5 min) after maximal incremental exercise testing reflects the coordinated cardiovascular autonomic adjustments, *i.e.,* the interplay between rapid cardiovagal reactivation and progressive sympathetic withdrawal on the sinus node—the autonomic balance (Cole et al., 1999; Okutucu et al., 2011). Spontaneous heart rate variability (HRV) assesses these autonomic adjustments based on the oscillations between consecutive heartbeats (R-R intervals) in different clinical conditions, such as throughout the recovery phase. HRV analysis is a suitable and reliable method for determining the relative strength of cardiovagal reactivation and sympathovagal balance on the sinus node (Malik et al., 2019).

Over the years, studies have consistently demonstrated the benefits of a physically active lifestyle, either based on structured exercise (*i.e.,* aerobic exercise), unstructured activities (*i.e.,* active commuting), or both, on HRR and HRV in promoting cardiovascular health (Alansare et al., 2018; Da Silva et al., 2015). Several studies have shown that regular aerobic exercise, characterized by continuous endurance training, has been widely recommended to improve cardiac autonomic function (Abolahrari-Shirazi et al., 2019; Kiss et al., 2016) and consequently to reduce cardiovascular morbimortality (Reimers, Knapp & Reimers, 2018).

However, a recent worldwide survey has shown that high-intensity exercise methods have been considered the leading trend in the fitness world, ranking within the top 10 exercise modalities most practiced since 2014 (Thompson, 2023). In this sense, based on a recent discussion about structured exercise concepts, fitness functional training (FFT) has been recognized as a high-intensity exercise training that combines different exercises (Ide et al., 2021) and incorporates multiple fitness domains (*e.g.*, endurance, resistance, and calisthenics) during the exercise sessions (Claudino et al., 2018). Thus, as an alternative to traditional aerobic exercises, FFT could potentially promote cardiovascular health to its practitioners (Garber et al., 2011; Martland et al., 2020). Additionally, considering the increasing demand for more time-efficient interventions and the demonstrated cardiovascular benefits of high-intensity training (Martland et al., 2020; Thompson, 2023), it is essential to investigate whether FFT can induce adaptations similar to or complementary with those from continuous endurance training, particularly regarding cardiac autonomic function. From a cardiac autonomic perspective, only one study has shown that FFT athletes exhibited impaired HRR compared to triathlon athletes following exercise (Morlin et al., 2023).

Although the authors reported impaired HRR in fitness functional training group (FFG), the cardiac autonomic adjustments during recovery remain unexplored in these practitioners. HRR analysis alone provides a limited estimate of autonomic adjustments, whereas post-exercise HRV offers a valid and reliable assessment of sympathovagal modulation during recovery (De Melo et al., 2022; Malik et al., 2019). Including post-exercise HRV analysis is essential for improving the understanding of cardiac autonomic adjustments induced by the training modality, offering new insights into previous studies and enabling a more precise characterization of cardiac autonomic control in FFT athletes.

Therefore, given the rising popularity of high-intensity training methods, exploring additional parameters such as HRV—examined less thoroughly in this context—is crucial. To our knowledge, this is the first study to simultaneously assess short-term HRR and HRV after maximal exercise testing in practitioners of different modalities. Thus, this approach may provide a more comprehensive understanding of post-exercise cardiac autonomic adjustments and contribute new evidence to existing literature.

Given the established benefits of endurance training on cardiac autonomic function, the lack of studies exploring the impact of FFT on HRR and HRV, and the global interest in the FFT method, it is crucial to evaluate the HRR and HRV in FFT practitioners in comparison to endurance practitioners and physically active individuals (*i.e.,* non-specific exercise training). This comparison may provide an initial understanding of FFT practices’ potential in the post-exercise cardiac autonomic adjustments following exercise. Thus, we hypothesized that practitioners of FFT have slower HRR and lower HRV than recreational endurance athletes and similar HRR and HRV to physically active men following maximal incremental exercise testing.

Therefore, this study aimed to compare the short-term HRR and HRV in men who are FFT recreational athletes with recreational athletes of endurance training and physically active men following maximal incremental exercise testing.

## Materials & Methods

### Study group and protocol

This observational analytical cross-sectional study with a convenience sampling procedure was carried out with fifty-three men (*n* = 53) with a median (quartiles) age of 32 (29–34) years old and a body mass index of 24 (22.8–26.1) kg/m^2^. Participants were eligible for inclusion if they were men, healthy (clinical examination), and between 20 and 40 years old. Exclusion criteria were non-sinus rhythm (*i.e.,* electrocardiogram), smoking, and under any medication**.** They were previously instructed to avoid stimulants and alcoholic beverages for 24 h and to stop any physical activity for at least 48 h before the tests (Timón et al., 2019). All participants provided written informed consent, and the Institutional Ethical Committee on Human Research of the Faculty of Medicine, University of Brasilia, approved (PN:1.151.967) this study in compliance with the National Research Ethics System Guidelines and Declaration of Helsinki.

The sample was composed of three groups, according to their physical training modality: the fitness functional group (FFG: *n* = 15) was composed of recreational FFT athletes (*i.e.,* Crossfit) with the age of 31.6 ± 3.7 years old and a body mass index of 25.2 ± 1.97 kg/m^2^, the endurance training group (EG: *n* = 23) was composed of recreational triathlon athletes aged 32.7 ± 2.8 years old and a body mass index (BMI) of 23.6 ± 1.8 kg/m^2^, and a control group (CG: *n* = 15) of physically active subjects with a non-specific exercise routine but who achieved the minimum recommended physical activity for health, evaluated using the International Physical Activity Questionnaire (IPAQ) (Bull et al., 2020), with the ages of 29.7 ± 3.9 years old and a body mass index of 23.9 ± 2,8 kg/m^2^.

Neither recreational athletes’ group was in a specific training or pre-competition phase. The athletes’ evaluations occurred during their maintenance training phase. During the evaluation period, it was observed that the EG engaged in an average of two daily activity sessions, 7/6 days a week. The total training volume accumulated an average of ≈17 h of moderate-intensity exercises per week, and they have been engaging in endurance activities for an average of four years daily. The FFG had one daily session of activities, which included endurance, strength training, and calisthenics in an interval and high-intensity exercise. This exercise routine was performed six days a week, totaling roughly an average of 7 h per week with high-intensity exercise. They have been engaged in FFT activities for an average of 3.3 years daily. The exercise regime of the FFG is often commercially called CrossFit training. However, the discussion about the more appropriate nomenclature of the exercise regime is beyond our scope and may involve commercial brands that must be respected. Therefore, the characterization of this group was based on the exercise regime due to its potential relationships with specific physiological adaptations.

Initially, we collected information on lifestyle habits, conducted a clinical examination, and collected anthropometrical (body mass and height) and basic physiological data: HR, blood pressure, and respiratory rate (RR). A 12-lead electrocardiograph recording (WinCardio v.5.0—Micromed^^®^^-Brazil) followed this examination was performed in the supine position in a quiet laboratory room between 08:00 and 10:00 a.m., at ambient temperature (21–24 °C) and relative humidity of 50 to 60%. After 20 min of rest in the supine position, the subjects were asked to actively adopt the orthostatic posture at the bedside. A valid 5-minute R-R interval series was recorded (short-term), following two minutes in this position, as described by Molina et al. (2016) and Molina et al. (2013). Prior to R-R interval recording, blood pressure measurement was conducted to verify the absence of hypotension in the orthostatic position, and the orthostatic stress test was performed as previously described (Molina et al., 2021). Following the pre-protocol instruction to achieve the new body position in no more than 10 s (Hnatkova et al., 2019).

Immediately after resting measures, a maximum treadmill-graded exercise test was conducted. During the 60 final seconds of the maximum voluntary effort and throughout the post-exercise active recovery protocol (Cole et al., 1999), a valid 1-min R-R interval series was recorded (ultra-short-term) in segments of 1 min, up to 5 min of recovery (Da Cruz et al., 2017; Goldberger et al., 2006).

### Heart rate variability analysis

The R-R interval series were recorded using a reliable Polar^®^ heart rate monitor (model RS800CX) with a sampling rate of 1,000 Hz (DerBarbosa et al., 2016). After recording, each series was transferred to a microcomputer for offline processing and analysis of R-R interval variability using Kubios HRV software (version 3.3.1, Kuopio, Finland).

Before processing the HRV data, all the tachogram series were visually reviewed to validate the presence of a sinus rhythm, identify non-sinus and artifacts, and assess the reliability of the signal. When spurious beats were detected, they were deleted from the tachogram series, along with their preceding and subsequent intervals, without adding new intervals. Eventually, outliers’ beats were also removed from the series. After that, an automated artifact/spurious beat identification and removal method was performed using a threshold method with a medium threshold. This method removes ectopic points, not exceeding 1% on the tachogram, keeping the physiological pattern as previously described (Tarvainen et al., 2014).

Qualified R-R interval series were highly steady and stationary (*i.e.,* resting orthostatic position) as estimated by the percent differences of the means and the standard deviations between the three divided series segments. Therefore, at rest, the qualified R-R interval tachograms were steady and stationary as determined by the percentage differences in the means and standard deviations among the three segmented series. During and following the treadmill incremental exercise, a valid 1-min R-R interval series was recorded (*i.e.,* ultra-short-term HRV measurement) in segments of 1 min, from the 60 final seconds of maximum voluntary effort up to 5 min of recovery as previously described (Da Cruz et al., 2017; Goldberger et al., 2006).

The variability of the R-R interval series was analyzed using Poincare’s Plot analysis, which is a geometric method that visually represents the correlation of successive R-R intervals. This method allows for a dynamic analysis of the R-R interval variability (Tulppo et al., 1996). The graphical representation takes the form of an ellipse, which can yield two quantitative indicators: SD1, an instantaneous index of the R-R series variability representing cardiovagal modulation, and SD2, a measure of the length of the plot influenced by both parasympathetic and sympathetic tones, reflecting overall variability (De Vito et al., 2002; Gomes et al., 2018; Shaffer & Ginsberg, 2017; Shi, Hu & Yu, 2018; Tulppo et al., 1998). The present study applied only the SD1 and SD2 indexes for cardiac autonomic analysis. Additionally, the variability of the R-R interval series was analyzed using frequency-domain analysis. For this analysis, the segments were filtered using a Hanning window and then processed with autoregressive modeling, employing a fixed order of 16, to convert the oscillating components of the signal into a power spectrum. This spectrum includes very-low-frequency (VLF, 0–0.04 Hz), low-frequency (LF, 0.04–0.15 Hz), and high-frequency (HF, 0.15–0.50 Hz) bands. The frequency domain indices calculated are: (a) total power spectral area (0–0.50 Hz) (TP), which indicates overall autonomic activity; (b) absolute power of the low- and high-frequency bands, which are respectively surrogates of combined sympathetic and parasympathetic activity and exclusive parasympathetic activity; The frequency-domain index calculated includes heart rate/LF (HR/LF), which has recently been identified as an indicator of sympathetic activity in HRV (Tanoue et al., 2022).

### Cardio-pulmonary exercise test and post-exercise heart rate recovery

The cardiopulmonary exercise test consisted of a graded treadmill exercise test according to the individualized ramp protocol (Riebe et al., 2018). The test started at a speed of three km/h and a 2.5% grade; the grade remained constant throughout the test, and the speed was increased gradually until volitional fatigue set in.

All tests were performed between 8 and 12 min on a conventional treadmill (ATL, Imbrasport, Brazil). Maximal oxygen uptake (VO_2_max) was obtained as described by Riebe et al. (2018), and the maximal respiratory exchange ratio (R), which is the ratio of carbon dioxide production to oxygen consumption, was measured by pulmonary gas exchange using the Ergoespirometer—Cortex Metalyzer 3B (Biophysik, Leipzig, Germany).

Following the exercise test, the treadmill was set at a walking speed of 2.4 km/h, and the grade was maintained at 2.5% (Cole et al., 1999). Participants realized a post-exercise active cool-down for 5 min. This recovery protocol was adopted considering its technical feasibility, reproducibility, and frequent application in the clinical setting.

During active recovery, the absolute and relative values of HRR were assessed by the HR reduction from HR peak to HR at 1st, 2nd, 3rd, 4th, and 5th minutes throughout the recovery phase (Cole et al., 1999; Molina et al., 2016).

### Statistical analysis

The Shapiro–Wilk test analyzed and rejected the normality distribution hypothesis in some variables. Thus, based on a nonparametric distribution, the data were described as medians and 25- and 75-quartiles.

We applied the Generalized Linear Model (GLM) (Nelder & Wedderburn, 1972) to compare differences between groups for HRR and HRV in different functional conditions (*i.e.,* rest, exercise, and recovery). GLM has some essential advantages over regular ANOVAs and nonparametric analyses, especially in research with small sample numbers in which the ANOVA assumptions are rarely met. These advantages (including the possibility of using different distributions for the parameters of the dependent variable) are explored in detail in the works of De Melo et al. (2022) and Yu et al. (2022). The GLM model that better fits statistically (Gaussian or Gamma distribution) with the identity link function, using Akaike’s Information Criterion (AIC), was adopted to analyze the results. Specifically, for HRV outcomes (SD1, SD2, HR/LF), a Gamma distribution family with identity link was used at each 1-minute segment in the 5-minute recovery. For HRR outcomes (absolute and relative), early recovery segments were better fit by Gamma distribution family (1st minute), whereas later segments were better fit by Gaussian distribution family (2nd to 5th minute); the identity link was used in all models. The AIC is interpreted considering that smaller values are better (Mccullagh, Nelder & Mccullagh, 1989). *Post hoc* pairwise comparisons used the Least Significant Difference (LSD) test due to the small number of planned pairwise contrasts; primary inferences relied on GLM main effects with covariate adjustment. Effect sizes were estimated using standardized coefficients Beta (β). We also employed Spearman’s correlation to assess the influence of confounding variables (age, BMI, arterial blood pressure, resting orthostatic HR, peak HR, R during exercise, and VO_2_ max) on HRR and HRV during recovery phase. Therefore, the GLM model was adjusted (covariates) to compare group differences for these confounders when necessary. All statistical tests were two-sided with a significant level of ≤0.05.

The sample size of 42 participants required for this study, through G*Power 3.1 software, was calculated by adopting an alpha error level of 0.05, a beta error of 0.20 (power of 0.80), and a large effect size (0.50), which indicates a sample of 14 participants by groups to detect differences among groups. The statistical analysis and figures were performed using JAMOVI 2.3.28 and GraphPad Prism 9.4.0 software.

## Results

The median (extreme) arterial blood pressure during resting was CG: 122/80 (112/130, 68/88) mmHg, EG: 126/84 (100/130, 60/90) mmHg, and FFG: 126/80 (100/130, 70/90) mmHg, (*p* = 0.32 to 0.34) were within the normal range in all groups at rest.

Table 1 shows the anthropometric and functional variables before and during exercise. There are differences in age and BMI between groups (CG *vs.* EG; β =  − 2.92; 95% CI [−5.13 to −0.71]; *p* = 0.01), (EG *vs.* FFG; β =  − 1.65; 95% CI [−3.06 to −0.25]; *p* = 0.03), respectively. CG shows high HR compared to EG at the pre-exercise rest period, 74 (69, 78) bpm *versus* 64 (60, 70) bpm, β = 7.55; 95% CI [1.36–13.74]; *p* = 0.02. Cardiovagal activity at rest (SD1) was lower in CG 16.6 (13.4, 19.7) ms than in EG 23.8 (17.9, 30.1) ms, β =  − 9.47; 95% CI [−17.06 to −2.18]; *p* = 0.01 and then in FFG 26.7 (18.2, 37.8) ms, β =  − 9.76; 95% CI [−19.05 to −1.70]; *p* = 0.02. During the exercise testing, the total time (TT) in CG was higher 633 (567, 739) s than in EG 565 (547, 617) s, (β = 84.07; 95% CI [31.27–138.72]; *p* = 0.00) and higher than FFG 545 (507, 562) s, (β = 115.67; 95% CI [59.21–173.13]; *p* = 0.00). CG presented lower maximal oxygen uptake than EG (β =  − 4.8; 95% CI [−8.66 to −0.93]; *p* = 0.02) and higher sympathetic modulation (HR/LF) values than EG (β = 2801.99; 95% CI [1,598.37–5,020.69]; *p* < 0.01) and than FFG (β = 2,709.45; 95% CI [1,471.22–4,935.11]; *p* < 0.01) at the peak of exercise. The maximal respiratory exchange ratio (R) was greater in FFG than in CG (β = 0.14; 95% CI [0.08–0.19]; *p* = 0.00) and EG (β = 0.11; 95% CI [0.06–0.16]; *p* = 0.00).

**Table 1:** Median (25th, 75th percentiles) of anthropometrical and basic physiological variables before and during exercise in the control group (CG), endurance group (EG) and fitness functional group (FFG).

**Anthropometrical**	**CG (*n* = 15)**	**EG (*n* = 23)**	**FFG (*n* = 15)**	**P**	**P** ^*^
Age (years)	30 (27–32)	33 (31–35)	33 (29–34)	0.03	CG *vs.* EG = 0.01
BMI (kg/m^2^)	24.2 (22.1–25.9)	23.5 (22.4–24.6)	26 (23.5–26.5)	0.06	EG *vs.* FFG = 0.03
**Pre-exercise, rest** **orthostatic period**					
HR (bpm)	74 (69–78)	64 (60–70)	72 (63–77)	0.05	CG *vs.* EG = 0.02
RR (breaths/min)	14 (11–17)	15 (13–17)	15 (12–19)	0.66	
SD1 (ms)	16.6 (13.4–19.7)	23.8 (17.9–30.1)	26.7 (18.2–37.8)	0.03	CG *vs.* EG = 0.01 CG *vs.* FFG =0.02
SD2 (ms)	49.3 (40.5–62.4)	55.3 (45.4–75.7)	57.1 (50.5–61.6)	0.24	
HR/LF	0.11 (0.05–0.14)	0.05 (0.02–0.10)	0.06 (0.05–0.11)	0.40	
**Exercise period**					
HR_peak_ (bpm)	184 (180–190)	182 (179–186)	185 (177–188)	0.60	
VO_2__peak_ mL (kg min)^−1^	46.4 (40.8–51.7)	51.2 (47.8–53.7)	46.4 (44.4–50.6)	0.04	CG vs EG = 0.02
TT (seconds)	633 (567–739)	565 (547–617)	545 (507–562)	0.00	CG *vs.* EG = 0.00 CG *vs.* FFG = 0.00
SD1_peak_ (ms)	2.9 (2.5–3.4)	3.2 (2.4–3.6)	2.8 (2.5–3.0)	0.70	
SD2_peak_(ms)	2.4 (1.9–2.9)	2.2 (1.9–2.8)	2.1 (1.8–2.7)	0.38	
HR/LF_peak_	1455 (570–3342)	501 (389–661)	557(384–877)	0.00	CG *vs.* EG = 0.00 CG *vs.* FFG = 0.00
R	1.2 (1.11–1.21)	1.2 (1.18–1.23)	1.3 (1.23–1.36)	0.00	CG *vs.* FFG = 0.00 EG *vs.* FFG = 0.00

**Notes.**

BMI body mass index HR heart rate RR respiratory rate SD1 instantaneous index of the variability of the R-R series SD2 measures the length of the plot Poincare’s HF/LF ratio of heart rate to low rate VO2 oxygen uptake TT total time R respiratory exchange ratio p *p* value for Generalized Linear Model

* *p*-value statistically different (*p* ≤ 0.05) for least significant difference *post hoc* test.

**Figure 1:**
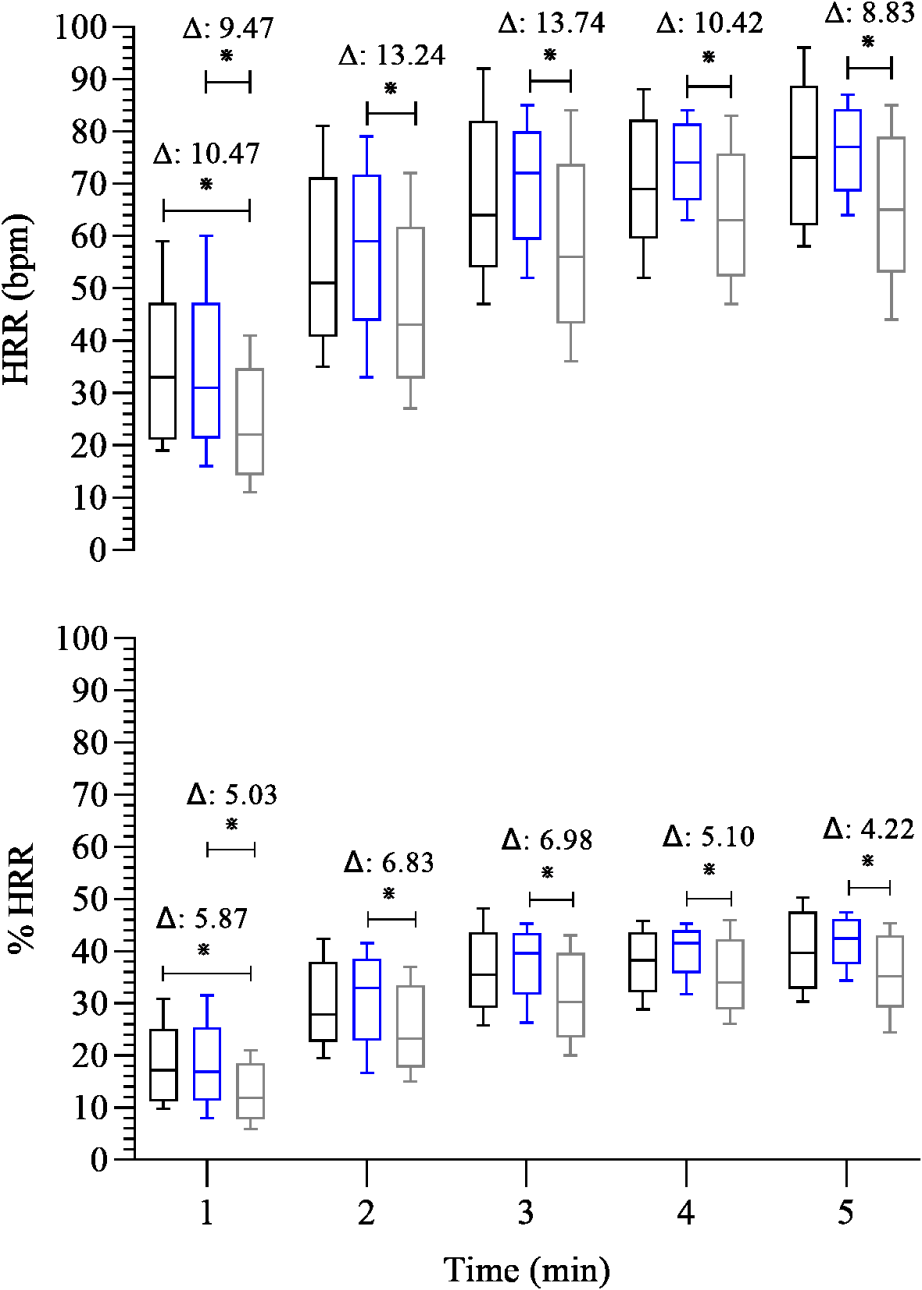
Comparison between-group of post-exercise chronotropic response. Boxplots represent the median (line), interquartile range (box), and whiskers indicating minimum and maximum values. All data points are included within these limits; therefore, no outliers appear as individual points. Median (25°, 75° percentiles) of the absolute (HRR) and relative (%HRR) recovery heart rate during the 5 min after the maximal exercise test in the control group—black box (*n* = 15) endurance group—blue box (*n* = 23) and the fitness functional group—gray box (*n* = 15). The groups were compared at each time point using the Generalized Linear Model analysis adjusted for covariates, followed by least significant difference *post hoc* tests. Axis values are reported as integers (no decimal places) for consistency across figures, while Δ values are shown with two decimal places. ^∗^: *p* ≤ 0.05. Δ: Difference in estimated means.

Before we compare group differences, we evaluated the influence of confounding variables on HRR and HRV during the recovery phase. We observed that age, BMI, TT, R, and HR/LF were significantly correlated with HRR, %HRR (r_s_ = from −0.32 to 0.43; *p* = 0.00–0.04). Therefore, all the following results were extracted from a multivariate GLM model adjusted for age, BMI, TT, R, and HR/LF, as specified in Methods. Thus, regarding the HRR (Fig. 1), FFG showed slower HRR than EG in all post-exercise time points (β =  − 13.74 to −8.83; 95% CI [−22.07 to −1.43]; *p* < 0.01 to 0.02). In addition, FFG also showed lower absolute HRR compared to CG at the 1st minute of recovery (β =  − 10.47; 95% CI [−21.19, −0.42]; *p* = 0.03). The same was observed for %HRR, whereas FFG showed a slow HRR in all post-exercise time points than EG (β =  − 6.98 to −4.22; 95% CI [−11.69 to −0.01] *p* < 0.01 to 0.05) and compared to CG at the 1st minute of recovery (β =  − 5.87; 95% CI [−11.87, −0.20]; *p* = 0.03).

**Figure 2:**
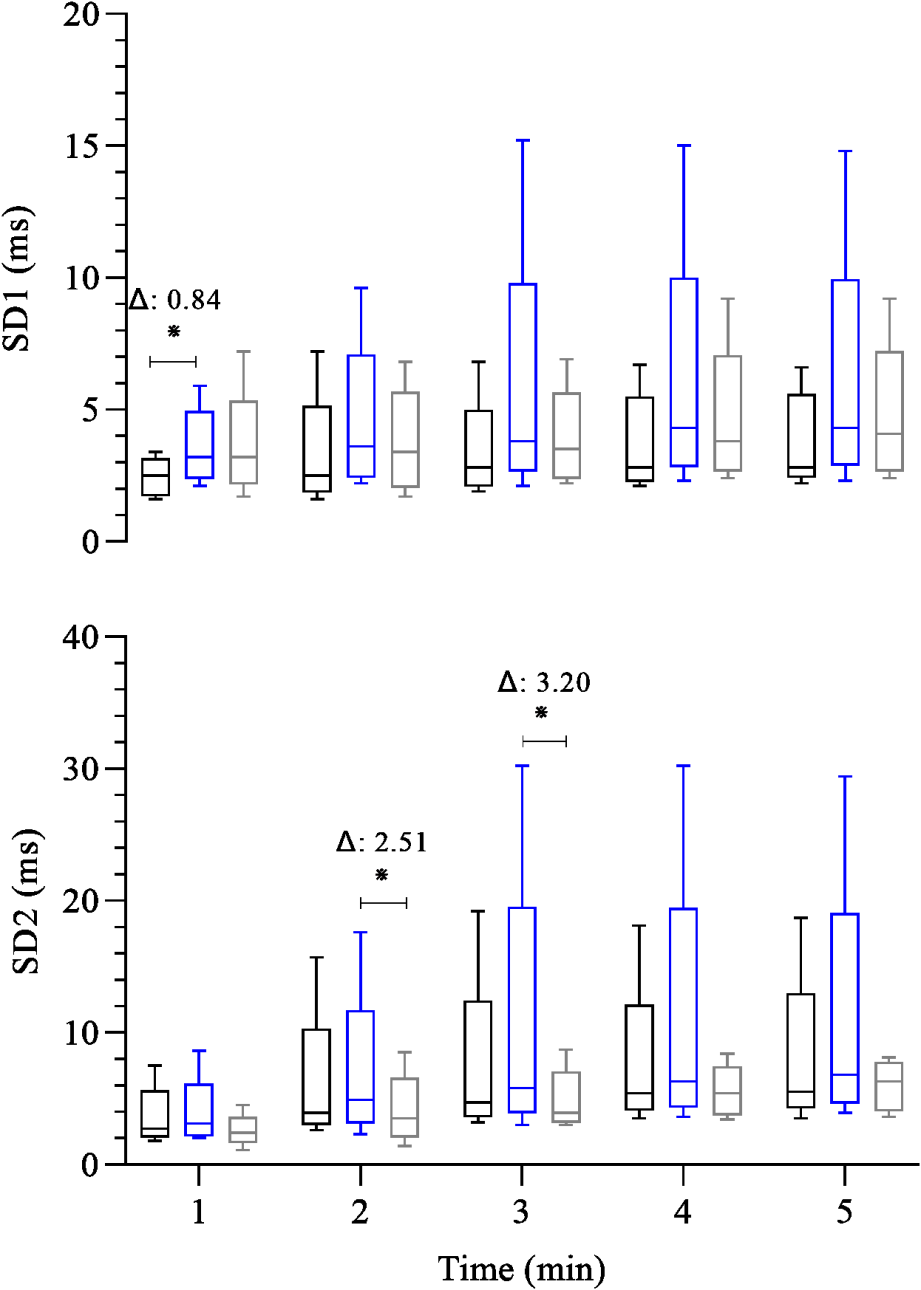
Comparison between-group of post-exercise autonomic response (SD1 and SD2 index). Boxplots represent the median (line), interquartile range (box), and whiskers indicating minimum and maximum values. All data points are included within these limits; therefore, no outliers appear as individual points. Median (25°, 75° percentiles) of the SD1 index and SD2 index (both expressed in milliseconds) analyses the heart rate variability during the 5 min after the maximal exercise test in the control group-black box (*n* = 15) endurance group-blue box (*n* = 23) and the fitness functional group-gray box (*n* = 15). The groups were compared at each time point using the Generalized Linear Model analysis adjusted for covariates, followed by least significant difference *post hoc* tests, ^∗^: *p* ≤ 0.05, Δ: Difference in estimated means.

Figure 2 shows the analysis of post-exercise heart rate variability among groups. Cardiovagal reactivation at 1st minute was significantly lower in CG than EG (CG: 2.5 (1.8, 3.0) ms *versus* EG: 3.2 (2.7, 4.0) ms, β =  − 0.84; 95% CI [−1.53, −0.12]; *p* = 0.02). In the other post-exercise time points, no significant difference exists among all groups throughout the recovery. Concerning the post-exercise overall cardiac autonomic modulation (SD2), at 2nd and 3rd minutes of recovery the FFG showed a reduced overall cardiac autonomic modulation compared to (EG, 4.9 (4.0, 5.8) *versus* FFG: 3.5 (2.7, 4.8) ms, β = 2.51; 95% CI [0.17–4.68]; *p* = 0.02) and (EG: 5.8 (4.8, 8.9) ms *versus* FFG: 3.9 (3.3, 5.5) ms, β = 3.20; 95% CI [0.28–6.13]; *p* = 0.03), respectively.

**Figure 3:**
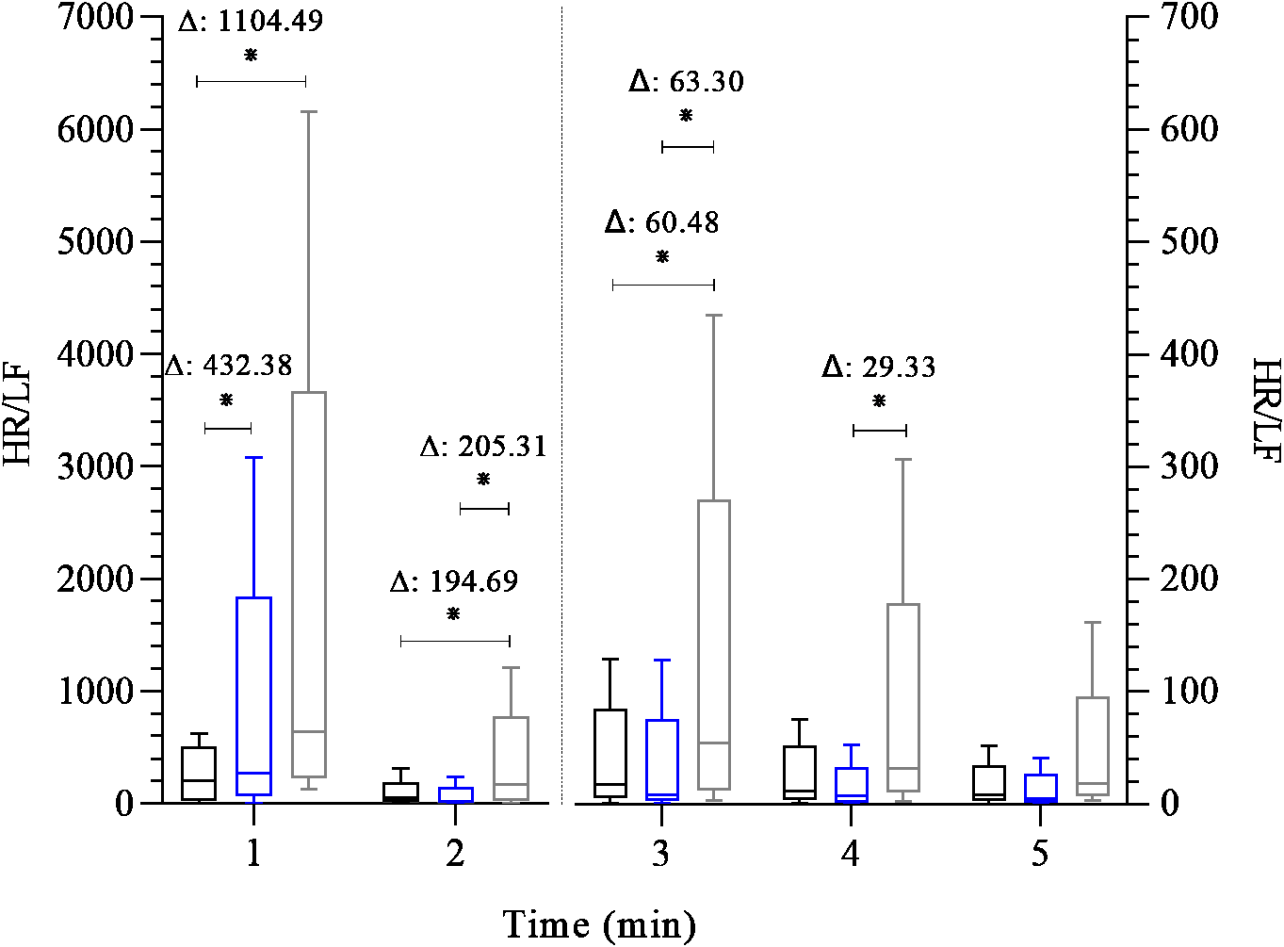
Comparison between groups of post-exercise autonomic response (HR/LF index). Boxplots represent the median (line), interquartile range (box), and whiskers indicating minimum and maximum values. All data points are included within these limits; therefore, no outliers appear as individual points. Median (25°, 75° percentiles) of the HR/LF index during the 5 min after the maximal exercise test in the control group—black box (*n* = 15) endurance group - blue box (*n* = 23) and the fitness functional group - gray box (*n* = 15). The groups were compared at each time point using the Generalized Linear Model analysis adjusted for covariates, followed by least significant difference post hoc tests. Axis values are reported as integers (no decimal places) for consistency across figures, while Δ values are shown with two decimal places. Two y-axes were used (left and right) due to large differences in HR/LF scale across time points. This approach allows visualization of early (1–2 min) and later (3–5 min) recovery distributions without losing quartile or whisker detail. ^∗^: *p* ≤ 0.05, Δ: Difference in estimated means. Note: the HR/LF index is dimensionless, derived from the ratio between HR (bpm) and LF power (ms^2^), and is used comparatively as a sympathetic marker.

Figure 3 shows the analysis of the HR/LF variable, indicative of sympathetic activity, throughout the post-exercise recovery. Differences were observed among groups at the 1st minute of recovery, with both FFG and EG showing higher sympathetic activity compared to CG (β = 432.38 to 1,104.49; 95% CI [165.73–1,871.29] *p* < 0.01). In addition, FFG showed higher sympathetic activity in the 2nd and 3rd minutes of recovery than CG (β = 60.48 to 194.69; 95% CI [−1.29 to 352.01]; *p* = 0.01 to 0.05) and than EG (β = 63.30 to 205.31; 95% CI [6.51–354.45]; *p* < 0.01 to 0.03). At the 4th minute, the FFG displayed a higher sympathetic activity value than EG (β = 29.33; 95% CI [2.95–66.79]; *p* = 0.05). No differences were observed among groups at 5th minute of recovery.

## Discussion

Our results showed new and significant findings regarding this observational analytical cross-sectional study, in which we compared the HRR and HRV in FFT recreational athletes, endurance recreational athletes, and a physically active control group, following maximal incremental treadmill exercise testing.

Considering the chronotropic response, we observed a consistently low HRR in FFG throughout all post-exercise time points compared to EG (Fig. 1). In addition, FFG also exhibited lower absolute and relative HRR than CG during the first minute of recovery. Regarding cardiac autonomic function following exercise, the cardiovagal reactivation (SD1) was lower at the 1st minute of recovery in the CG than in the EG (Fig. 2) and was similar between the FFG and EG. Interestingly, no differences (*p* > 0.05) in cardiovagal reactivation were observed among groups from the 2nd minute of recovery to its end. Regarding the overall cardiac autonomic modulation (SD2), FFG showed significantly reduced autonomic modulation in the 2nd and 3rd minutes of recovery compared to EG. These differences in the cardiac autonomic adjustments associated with low HRR and similar cardiovagal reactivation following exercise, particularly for FFG, may suggest persistent sympathetic activity compared to the EG (Figs. 2 and 3). At the first minute of recovery, both FFG and EG presented a higher indication of sympathetic activity (HR/LF) compared to CG. However, FFG consistently showed the greatest sympathetic activity throughout the subsequent recovery period. This was evident from the 2nd to the 4th minute, when FFG showed higher values of sympathetic activity than CG and EG at the 2nd and 3rd minutes, and higher than EG at the 4th minute.

Therefore, these findings suggest that EG displayed a faster and more efficient chronotropic response during the recovery phase (restoration to the initial baseline level) following the maximal treadmill exercise test than FFG. In other words, our findings suggested that the decrement of HRR was reduced in FFG compared to EG due to a persistent coactivation of both autonomic branches throughout the recovery phase (5 min).

Although the HRR and its cardiac autonomic modulation share a common physiological functional basis, their intricate interplay and impact on each other during the recovery phase still need to be fully understood. Therefore, the complete portrait of the complexity of all mechanisms involved in the cardiac autonomic function and HR dynamics can only be conjectured. In this scenario, our study adds to previous knowledge (Morlin et al., 2023) by showing different cardiac autonomic modulation according to the exercise training modality.

As established, HRR assesses the rate at which HR decreases following exercise testing (Goldberger et al., 2006; Imai et al., 1994) reflects the coordinated interaction between rapid cardiovagal reactivation (inhibitory activity) and slow and progressive sympathetic withdrawal (Michael, Graham & Davis, 2017). So, the coactivation of both branches occurs several minutes immediately after the exercise. Consequently, hyperactivity of both autonomic branches is present for at least 1 min immediately following the exercise (Tulppo et al., 2011). Interestingly, despite this, the primary autonomic adjustment at this stage (1st minute) appears to rely on cardiovagal reactivation (Goldberger et al., 2006; Imai et al., 1994); the FFG showed a lower HRR (*P* < 0.05) in the first minute than CG and EG despite the similar cardiovagal reactivation (*P* > 0.05) compared to CG and EG. Thus, one possibility is that the cardiovagal reactivation (inhibitory capacity) was less effective in reducing sympathetic excitatory stimulus toward a cardiovagal modulation predominance or a slow sympathetic withdrawal (excitatory capacity) in the same recovery phase. This possibility can be mainly reinforced by similar HR, cardiovagal activity, and sympathetic activity between FFG and EG at the end of the exercise (Table 1). During the 1st minute of recovery, FFG exhibited higher sympathetic activity (HR/LF) compared to CG. Following the 2nd minute of recovery, our observation is further supported by reduced overall cardiac autonomic modulation (*i.e.,* low modulation) in FFG at two time points (2nd and 3rd minutes) compared to EG, along with higher sympathetic activity compared to both groups at the 2nd and 3rd minutes of recovery. In addition, FFG showed elevated HR/LF at the 4th minute compared to EG.

Hence, this observation reinforces the hypothesis that FFG displayed more persistent sympathetic activity than the EG throughout the recovery phase, despite the same degree of cardiovagal reactivation (SD1) observed among the groups during this period. Similar SD1, despite elevated HR/LF in FFG, may reflect compensatory vagal reactivation during sustained sympathetic outflow. This apparent paradox may reflect an accentuated sympathovagal interaction during recovery, as described by Tulppo et al. (2011), where individuals with higher post-exercise sympathetic outflow also exhibit augmented vagal modulation. In such cases, SD1 may return to baseline levels despite sustained sympathetic activity due to compensatory vagal reactivation intended to restore cardiac stability. Although the present study design does not allow for a cause-and-effect analysis, our results provide a detailed description of the HRR phenomenon and its inherent cardiac autonomic function across three different exercise regimes. A possible explanation for a lower HRR, reduced overall cardiac autonomic function modulation, and high sympathetic activity observed in the FFG compared to CG and EG is the maintenance of metaboreflex activity during the recovery phase (O’leary, 1993), which can reduce the speed of HRR (Ba et al., 2009; Boyes et al., 2023) and the overall cardiac modulation (Tulppo et al., 2011). Thus, persistent sympathetic activity in FFG may reflect metaboreflex activation (Boyes et al., 2023), but longitudinal studies are needed to confirm this hypothesis. In addition, another potential explanation for the autonomic difference among groups could be arterial stiffness and autonomic cardiac function mediated by baroreflex (Virtanen et al., 2003). Recently, studies have reported a delay in the baroreflex response across different modalities, and arterial stiffness seems to be linked to this delay (De Paula et al., 2019; Nakamura & Muraoka, 2021). In this context, a systematic review has shown that endurance training positively affects arterial stiffness (Ashor et al., 2015). However, studies have found no changes in arterial stiffness after resistance exercise (Grigoriadis et al., 2020; Zuo et al., 2022), and some research suggests that high-intensity interval training might positively influence arterial stiffness (Hasegawa et al., 2018; Jalaludeen et al., 2020); however, this remains questionable (Holloway, Roche & Angell, 2018). Zuo et al. (2022) observed reductions in systemic arterial stiffness following traditional and functional resistance training in healthy young adults. In contrast, Hasegawa et al. (2018) reported no effects of resistance exercise on central arterial stiffness and nitric oxide bioavailability. These differences may result from varying measurement methods (CAVI *vs.* PWV), training protocols, and participant characteristics. While Zuo et al. (2022) focused on systemic outcomes, Hasegawa et al. (2018) examined endothelial molecular pathways, suggesting that the vascular response to resistance training is diverse and context-dependent. In 2022, a review highlighted that exercise benefits may depend on participants’ training mode, intensity, duration, and age (Liu et al., 2022). Therefore, further research is needed to clarify the effects of high-intensity interval training on autonomic function and arterial stiffness. Thus, as FFT incorporates high-intensity endurance, resistance, and calisthenics exercises, the lack of studies evaluating the effect of this type of exercise on arterial stiffness in healthy adults is a significant gap in current research. Our data support the hypothesis that FFT might be associated with lower benefits on arterial stiffness adaptation. This complex interplay of factors, combined with the lack of comprehensive studies, makes this issue a challenging and intriguing area for further exploration. Thus, it is still an open, incompletely explored issue.

Lastly, another possibility for our results is that 48 h of resting could not be sufficient to settle HRR and its variability in FFG compared to EG. Studies have shown that high-intensity intermittent exercise increases sympathetic predominance and affects cardiac autonomic modulation, reducing recovery/reactivity as a function of time (Cabral-Santos et al., 2016; Castrillón et al., 2017), which may support this possibility.

Our results follow a recent study published by Morlin et al. (2023), which showed that individuals who practice FFT as a primary exercise mode exhibited an impaired HRR following exercise compared to endurance athletes. The authors hypothesized that low HRR could be explained by lower cardiovagal reactivation and/or persistent sympathetic activity in FFG. Indeed, our study corroborates with Morlin’s research and adds that the lower chronotropic response observed throughout the recovery phase may be associated with high sympathetic activity instead of low cardiovagal reactivation, and therefore, the low HRR observed may be associated with a persistent coactivation of both autonomic branches throughout the recovery phase in practitioners of FFT. In addition, in the study by Kaikkonen, the authors suggest that the immediate recovery of HRR (5 min) after high-intensity exercise may be more a result of diminished sympathetic modulation and less vagal reactivation (Kaikkonen et al., 2024). In this context, a recent study (Boyes et al., 2023) demonstrated that the sympathoexcitation caused by muscle metaboreflex activation attenuated the HRR (5min) following moderate-vigorous intensity, dynamic exercise (Boyes et al., 2023).

Hence, from a clinical perspective, even though all groups displayed a high oxygen uptake peak, faster HRR at the 1st minute, and the same cardiovagal reactivation from the 2nd to 5th minute of recovery, the FFG showed chronotropic impairment from the 1st to 5th minute of recovery compared to EG, low overall cardiac autonomic modulation than EG, and high activity of HR/LF throughout the recovery phase compared to EG from the 2nd to 4th minute of recovery.

These results can be considered a novel and important finding in the sports field, considering the growth of FFT practitioners worldwide. In this sense, a better understanding of the specific physiological adaptations to exercise in this group is an essential task for exercise physiologists and coaches.

Therefore, our study may provide initial information regarding the HRR and its autonomic adjustments after exercise in recreational athletes who use FFT as their primary exercise mode, compared to recreational endurance athletes and physically active men with non-specific exercise routines. In addition, these findings should be considered in the context of the growing popularity of FFT and the lack of studies that analyze FFT as a practice for promoting cardiovascular health.

Despite the strength of our methodological approach, which includes a physically active control group instead of the traditional control group composed of inactive participants and a robust statistical analysis, it is important to note that the use of an active control group, rather than sedentary group, may limit the generalizability of our findings compared to conventional exercise-training studies. The current study has limitations, such as the cross-sectional design, a non-random sample, and the lack of individual control of training sessions associated with checking possible signs of overtraining. Although age was methodologically controlled through inclusion criteria (participants aged 20–40 years), and both age and BMI were adjusted for in the statistical models, the possibility of residual confounding related to these variables cannot be completely ruled out. On the other hand, our results should be carefully observed from the cardiac autonomic perspective. Indeed, the low HRR in FFG compared to EG, and similar cardiovagal reactivation among groups throughout recovery, suggest a persistent coactivation of both autonomic branches on the sinus node in FFG. This may be linked to altered moment-to-moment cardiovascular function adaptation, reflecting poor homeostasis and vulnerability to functional disturbances and diseases (Cadegiani, Kater & Gazola, 2019; Jacob et al., 2020; Tibana et al., 2022; Timón et al., 2019).

It is also important to consider that the EG had a higher weekly training volume of moderate-intensity exercise. In contrast, the FFG engaged in lower-volume but higher-intensity exercise. This may have influenced the autonomic recovery patterns observed. Cabral-Santos et al. (2016) reported delayed parasympathetic reactivation following high-intensity intermittent exercise compared to moderate-intensity continuous training. However, their study involved passive recovery over 60 min, with differences emerging only after 20 min. In contrast, our protocol used active recovery and a shorter 5-minute window, which may elicit distinct autonomic responses. These methodological differences restrict direct comparison, but they emphasize the importance of exercise intensity, volume, and recovery type in evaluating post-exercise autonomic regulation. It is likely that modality, intensity, and volume all contributed to recovery kinetics; however, our cross-sectional design cannot isolate their independent effects. Therefore, considering our findings and the popularity of FFT, it is crucial to recognize that many potential FFT practitioners may have subclinical and asymptomatic cardiovascular conditions that need to be considered when the exercise regimen could be associated with delayed recovery.

Therefore, we cannot establish a cause-and-effect relationship even though we controlled the participants’ weekly exercise volume, and all participants were clinically evaluated and considered fit to carry out the present research. Hence, new studies should be conducted to progress even more in the understanding of the cardiovascular response and safety of FFG training. Additionally, the results cannot be generalized to men and women of different ages who engage in FFT at an athletic level.

## Conclusions

This study showed low HRR in recreational athletes of FFT compared to recreational endurance athletes. From the cardiac autonomic perspective, there is no difference in cardiovagal reactivation from the second minute to its end among groups. However, low overall cardiac modulation and high sympathetic activity were observed throughout recovery in FFG compared to other groups. Lastly, our results suggest an association with persistent coactivation of both autonomic branches following maximal exercise testing in the recreational athletes of FFT compared to the other two groups. These findings describe autonomic recovery patterns and are not designed to adjudicate cardiovascular risk.

##  Supplemental Information

10.7717/peerj.20335/supp-1 Supplemental Information 1 Raw Data

10.7717/peerj.20335/supp-2 Supplemental Information 2 STROBE

10.7717/peerj.20335/supp-3 Supplemental Information 3 Medians (25th, 75th percentiles), adjusted β coefficients (95% CI), and p-values for post-exercise chronotropic and autonomic responses in the control group (CG), endurance group (EG), and fitness functional group (FFG) Values for the control group (CG), endurance group (EG), and fitness functional group (FFG) are presented as medians (25th, 75th percentiles). Group comparisons were performed using a Generalized Linear Model (GLM) adjusted for age, BMI, total exercise time (TT), respiratory exchange ratio (R), and HR/LF at peak exercise. β (95% CI): difference between estimated means with 95% confidence interval. *p*, *p*-value for least significant difference *post hoc* test; *, p-value statistically different (*p* ≤ 0.05) for least significant difference *post hoc* test; HRR: absolute heart rate recovery; % HRR, relative heart rate recovery; SD1, instantaneous index of R-R series variability; SD2, measures the length from the Poincaré plot; HR/LF, heart rate/low-frequency ratio.

## Additional Information and Declarations

### Data Availability

The following information was supplied regarding data availability: The raw data are available in the Supplementary File.

## References

[ref-1] Abolahrari-Shirazi S, Kojuri J, Bagheri Z, Rojhani-Shirazi Z (2019). Effect of exercise training on heart rate variability in patients with heart failure after percutaneous coronary intervention. Journal of Biomedical Physics and Engineering.

[ref-2] Alansare A, Alford K, Lee S, Church T, Jung HC (2018). The effects of high-intensity interval training *vs.* moderate-intensity continuous training on heart rate variability in physically inactive adults. International Journal of Environmental Research and Public Health.

[ref-3] Ashor AW, Lara J, Siervo M, Celis-Morales C, Oggioni C, Jakovljevic DG, Mathers JC (2015). Exercise modalities and endothelial function: a systematic review and dose-response meta-analysis of randomized controlled trials. Sports Medicine.

[ref-4] Ba A, Delliaux S, Bregeon F, Levy S, Jammes Y (2009). Post-exercise heart rate recovery in healthy, obeses, COPD subjects: relationships with blood lactic acid and PaO2 levels. Clinical Research in Cardiology.

[ref-5] Barbosa MP da C de R, Da Silva NT, De Azevedo FM, Pastre CM, Vanderlei LCM (2016). Comparison of Polar^®^ RS800G3™ heart rate monitor with Polar^®^ S810i™ and electrocardiogram to obtain the series of RR intervals and analysis of heart rate variability at rest. Clinical Physiology and Functional Imaging.

[ref-6] Bellenger CR, Fuller JT, Thomson RL, Davison K, Robertson EY, Buckley JD (2016). Monitoring athletic training status through autonomic heart rate regulation: a systematic review and meta-analysis. Sports Medicine.

[ref-7] Boyes NG, Mannozzi J, Rapin N, Alvarez A, Al-Hassan M-H, Lessanework B, Lahti DS, Olver TD, O’leary DS, Tomczak CR (2023). Augmented sympathoexcitation slows post-exercise heart rate recovery. Journal of Applied Physiology.

[ref-8] Bull FC, Al-Ansari SS, Biddle S, Borodulin K, Buman MP, Cardon G, Carty C, Chaput J-P, Chastin S, Chou R, Dempsey C, Dipietro L, Ekelund U, Firth J, Friedenreich CM, Garcia L, Gichu M, Jago R, Katzmarzyk T, Lambert E, Leitzmann M, Milton K, Ortega FB, Ranasinghe C, Stamatakis E, Tiedemann A, Troiano RP, Van Der Ploeg HP, Wari V, Willumsen JF (2020). World Health Organization 2020 guidelines on physical activity and sedentary behaviour. British Journal of Sports Medicine.

[ref-9] Cabral-Santos C, Giacon TR, Campos EZ, Gerosa-Neto J, Rodrigues B, Vanderlei LC, Lira FS (2016). Impact of high-intensity intermittent and moderate-intensity continuous exercise on autonomic modulation in young men. International Journal of Sports Medicine.

[ref-10] Cadegiani FA, Kater CE, Gazola M (2019). Clinical and biochemical characteristics of high-intensity functional training (HIFT) and overtraining syndrome: findings from the EROS study (The EROS-HIFT). Journal of Sports Sciences.

[ref-11] Castrillón CIM, Miranda RAT, Cabral-Santos C, Vanzella LM, Rodrigues B, Vanderlei LCM, Lira FS, Campos EZ (2017). High-intensity intermittent exercise and autonomic modulation: effects of different volume sessions. International Journal of Sports Medicine.

[ref-12] Claudino JG, Gabbett TJ, Bourgeois F, Souza H de S, Miranda RC, Mezêncio B, Soncin R, Cardoso Filho CA, Bottaro M, Hernandez AJ, Amadio AC, Serrão JC (2018). CrossFit overview: systematic review and meta-analysis. Sports Medicine - Open.

[ref-13] Cole CR, Blackstone EH, Pashkow FJ, Snader CE, Lauer MS (1999). Heart-rate recovery immediately after exercise as a predictor of mortality. The New England Journal of Medicine.

[ref-14] Da Cruz CJG, Rolim P da S, Pires D de S, Mendes CMO, De Paula GM, Porto LGG, Garcia GL, Molina GE (2017). Reliability of heart rate variability threshold and parasympathetic reactivation after a submaximal exercise test. Motriz: Revista de Educacão Física̧.

[ref-15] Da Silva P, De Oliveira NA, Silveira H, Mello RGT, Deslandes AC (2015). Heart rate variability indexes as a marker of chronic adaptation in athletes: a systematic review. Annals of Noninvasive Electrocardiology: The Official Journal of the International Society for Holter and Noninvasive Electrocardiology, Inc.

[ref-16] Daanen HAM, Lamberts RP, Kallen L, Jin A, Van Meeteren NLU (2012). A systematic review on heart-rate recovery to monitor changes in training status in athletes. International Journal of Sports Physiology and Performance.

[ref-17] De Melo MB, Daldegan-Bueno D, Menezes Oliveira MG, De Souza AL (2022). Beyond ANOVA and MANOVA for repeated measures: advantages of generalized estimated equations and generalized linear mixed models and its use in neuroscience research. European Journal of Neuroscience.

[ref-18] De Paula T, Neves MF, DaSilva Itaborahy A, Monteiro W, Farinatti P, Cunha FA (2019). Acute effect of aerobic and strength exercise on heart rate variability and baroreflex sensitivity in men with autonomic dysfunction. Journal of Strength and Conditioning Research.

[ref-19] De Vito G, Galloway SDR, Nimmo MA, Maas P, Mcmurray JJV (2002). Effects of central sympathetic inhibition on heart rate variability during steady-state exercise in healthy humans. Clinical Physiology and Functional Imaging.

[ref-20] Garber CE, Blissmer B, Deschenes MR, Franklin BA, Lamonte MJ, Lee I-M, Nieman DC, Swain DP, American College of Sports Medicine, American College of Sports Medicine position stand (2011). Quantity and quality of exercise for developing and maintaining cardiorespiratory, musculoskeletal, neuromotor fitness in apparently healthy adults: guidance for prescribing exercise. Medicine and Science in Sports and Exercise.

[ref-21] Goldberger JJ, Le FK, Lahiri M, Kannankeril J, Ng J, Kadish AH (2006). Assessment of parasympathetic reactivation after exercise. American Journal of Physiology-Heart and Circulatory Physiology.

[ref-22] Gomes RL, Vanderlei LC, Garner DM, Santana MD, De Abreu LC, Valenti VE (2018). Poincaré plot analysis of ultra-short-term heart rate variability during recovery from exercise in physically active men. The Journal of Sports Medicine and Physical Fitness.

[ref-23] Grigoriadis G, Rosenberg AJ, Lefferts WK, Wee SO, Schroeder EC, Baynard T (2020). Similar effects of acute resistance exercise on carotid stiffness in males and females. International Journal of Sports Medicine.

[ref-24] Hasegawa N, Fujie S, Horii N, Miyamoto-Mikami E, Tsuji K, Uchida M, Hamaoka T, Tabata I, Iemitsu M (2018). Effects of different exercise modes on arterial stiffness and nitric oxide synthesis. Medicine and Science in Sports and Exercise.

[ref-25] Hnatkova K, šišáková M, Smetana P, Toman O, Huster KM, Novotný T, Schmidt G, Malik M (2019). Sex differences in heart rate responses to postural provocations. International Journal of Cardiology.

[ref-26] Holloway K, Roche D, Angell P (2018). Evaluating the progressive cardiovascular health benefits of short-term high-intensity interval training. European Journal of Applied Physiology.

[ref-27] Ide BN, Silvatti AP, Marocolo M, Santos CPC, Silva BVC, Oranchuk DJ, Mota GR (2021). Is there any non-functional training? A conceptual review. Frontiers in Sports and Active Living.

[ref-28] Imai K, Sato H, Hori M, Kusuoka H, Ozaki H, Yokoyama H, Takeda H, Inoue M, Kamada T (1994). Vagally mediated heart rate recovery after exercise is accelerated in athletes but blunted in patients with chronic heart failure. Journal of the American College of Cardiology.

[ref-29] Jacob N, Novaes JS, Behm DG, Vieira JG, Dias MR, Vianna JM (2020). Characterization of hormonal, metabolic, inflammatory responses in CrossFit^®^ training: a systematic review. Frontiers in Physiology.

[ref-30] Kaikkonen P, Pasanen K, Parkkari J, Mustakoski I, Vasankari T, Leppänen M (2024). Recovery of heart rate and heart rate variability after a maximal cardiopulmonary exercise test in novice female runners. European Journal of Applied Physiology.

[ref-31] Kiss O, Sydó N, Vargha P, Vágó H, Czimbalmos C, Édes E, Zima E, Apponyi G, Merkely G, Sydó T, Becker D, Allison TG, Merkely B (2016). Detailed heart rate variability analysis in athletes. Clinical Autonomic Research.

[ref-32] Liu W-L, Lin Y-Y, Mündel T, Chou C-C, Liao Y-H (2022). Effects of acute interval exercise on arterial stiffness and cardiovascular autonomic regulatory responses: a narrative review of potential impacts of aging. Frontiers in Cardiovascular Medicine.

[ref-33] Malik M, Hnatkova K, Huikuri HV, Lombardi F, Schmidt G, Zabel M (2019). CrossTalk proposal: heart rate variability is a valid measure of cardiac autonomic responsiveness. The Journal of Physiology.

[ref-34] Martland R, Mondelli V, Gaughran F, Stubbs B (2020). Can high-intensity interval training improve physical and mental health outcomes? A meta-review of 33 systematic reviews across the lifespan. Journal of Sports Sciences.

[ref-35] Mccullagh P, Nelder JA, Mccullagh M (1989). Generalized linear models, Second Edition: 37. 2nd ed. edição.

[ref-36] Michael S, Graham KS, Davis GM (2017). Cardiac autonomic responses during exercise and post-exercise recovery using heart rate variability and systolic time intervals—a review. Frontiers in Physiology.

[ref-37] Molina GE, Da Cruz CJG, Fontana KE, Soares EMKVK, Porto LGG, Junqueira LF (2021). Post-exercise heart rate recovery and its speed are associated with cardiac autonomic responsiveness following orthostatic stress test in men. Scandinavian Cardiovascular Journal.

[ref-38] Molina GE, Fontana KE, Porto LGG, Junqueira LF (2016). Post-exercise heart-rate recovery correlates to resting heart-rate variability in healthy men. Clinical Autonomic Research.

[ref-39] Molina GE, Porto LGG, Fontana KE, Junqueira LF (2013). Unaltered R-R interval variability and bradycardia in cyclists as compared with non-athletes. Clinical Autonomic Research.

[ref-40] Morlin MT, Da Cruz CJG, Guimarães FER, Da Silva RAS, Porto LGG, Molina GE (2023). High-intensity interval training combined with different types of exercises on cardiac autonomic function. an analytical cross-sectional study in CrossFit^®^ athletes. International Journal of Environmental Research and Public Health.

[ref-41] Nakamura N, Muraoka I (2021). Effects of greater central arterial stiffness on cardiovagal baroreflex sensitivity in resistance-trained men. Sports Medicine - Open.

[ref-42] Nelder JA, Wedderburn RWM (1972). Generalized linear models. Journal of the Royal Statistical Society. Series A (General).

[ref-43] Okutucu S, Karakulak UN, Aytemir K, Oto A (2011). Heart rate recovery: a practical clinical indicator of abnormal cardiac autonomic function. Expert Review of Cardiovascular Therapy.

[ref-44] O’leary DS (1993). Autonomic mechanisms of muscle metaboreflex control of heart rate. Journal of Applied Physiology.

[ref-45] Qiu S, Cai X, Sun Z, Li L, Zuegel M, Steinacker JM, Schumann U (2017). Heart rate recovery and risk of cardiovascular events and all-cause mortality: a meta-analysis of prospective cohort studies. Journal of the American Heart Association.

[ref-46] Reimers AK, Knapp G, Reimers C-D (2018). Effects of exercise on the resting heart rate: a systematic review and meta-analysis of interventional studies. Journal of Clinical Medicine.

[ref-47] Riebe D, Ehrman JK, Liguori G, Magal M, Medicine, A. C. of S (2018). ACSM’s guidelines for exercise testing and prescription.

[ref-48] Shaffer F, Ginsberg J (2017). An overview of heart rate variability metrics and norms. Frontiers in Public Health.

[ref-49] Shi P, Hu S, Yu H (2018). Recovery of heart rate variability after treadmill exercise analyzed by lagged Poincaré plot and spectral characteristics. Medical & Biological Engineering & Computing.

[ref-50] Stanley J, Peake JM, Buchheit M (2013). Cardiac parasympathetic reactivation following exercise: implications for training prescription. Sports Medicine.

[ref-51] Tanoue Y, Komatsu T, Nakashima S, Matsuda T, Michishita R, Higaki Y, Uehara Y (2022). The ratio of heart rate to heart rate variability reflects sympathetic activity during incremental cycling exercise. European Journal of Sport Science.

[ref-52] Tarvainen MP, Niskanen J-P, Lipponen JA, Ranta-Aho O, Karjalainen PA (2014). Kubios HRV–heart rate variability analysis software. Computer Methods and Programs in Biomedicine.

[ref-53] Thompson WR (2023). Worldwide survey of fitness trends for 2023. ACSM’S Health & Fitness Journal.

[ref-54] Tibana RA, De Sousa Neto IV, De Sousa NMF, Dos Santos WM, Prestes J, Neto JHF, Dominski FH, Kennedy MD, Voltarelli FA (2022). Time-course effects of functional fitness sessions performed at different intensities on the metabolic, hormonal, BDNF responses in trained men. BMC Sports Science, Medicine & Rehabilitation.

[ref-55] Timón R, Olcina G, Camacho-Cardeñosa M, Camacho-Cardenosa A, Martinez-Guardado I, Marcos-Serrano M (2019). 48-hour recovery of biochemical parameters and physical performance after two modalities of CrossFit workouts. Biology of Sport.

[ref-56] Tulppo MP, Kiviniemi AM, Hautala AJ, Kallio M, Seppänen T, Tiinanen S, Mäkikallio TH, Huikuri H (2011). Sympatho-vagal interaction in the recovery phase of exercise: postexercise autonomic regulation. Clinical Physiology and Functional Imaging.

[ref-57] Tulppo MP, Mäkikallio TH, Seppänen T, Laukkanen RT, Huikuri H (1998). Vagal modulation of heart rate during exercise: effects of age and physical fitness. The American Journal of Physiology.

[ref-58] Tulppo MP, Mäkikallio TH, Takala TE, Seppänen T, Huikuri H (1996). Quantitative beat-to-beat analysis of heart rate dynamics during exercise. The American Journal of Physiology.

[ref-59] Virtanen R, Jula A, Kuusela T, Helenius H, Voipio-Pulkki L-M (2003). Reduced heart rate variability in hypertension: associations with lifestyle factors and plasma renin activity. Journal of Human Hypertension.

[ref-60] Yu Z, Guindani M, Grieco SF, Chen L, Holmes TC, Xu X (2022). Beyond t test and ANOVA: applications of mixed-effects models for more rigorous statistical analysis in neuroscience research. Neuron.

[ref-61] Zuo C, Li Q, Zhang L, Bo S (2022). Effects of 6-week traditional and functional resistance training on arterial stiffness and muscular strength in healthy young men. Frontiers in Physiology.

