# Supplementary material for: Delayed heart rate recovery and its variability in fitness functional training compared to endurance athletes: a cross-sectional analysis
Source: PeerJ. 2026 Jan 5;14:e20335. doi: 10.7717/peerj.20335 (PMC12782030; doi:10.7717/peerj.20335)
Supplement: Supplemental Information 2 [file peerj-14-20335-s002.docx]

STROBE Statement—Checklist of items that should be included in reports of ***cross-sectional studies***

1. **TITLE AND ABSTRACT**

Delayde heart rate recovery and its variability in Fitness Functional Training compared to Endurance athletes: A cross-sectional analysis.

The study investigated recovery heart rate and its variability in functional fitness training athletes, endurance athletes and physically active individuals. A cross-sectional study was carried out comparing the three groups through statistical analysis of their cardiovascular recovery metrics. (Manuscript section: lines 40 to 64).

### ****INTRODUCTION****

### Background / rationale: Recovery heart rate (HRR) is a powerful predictor of cardiovascular morbidity and mortality and an indicator of athletic performance. The HRR reflects coordinated cardiovascular autonomic adjustments. However, we did not find studies in the literature that compared HRR and its variability between functional fitness training athletes, endurance athletes, and physically active men. (Manuscript section: lines 71 to 113).

### Objetivos: This study aimed to compare the short-term HHR and HRV in men who are FFT recreational athletes with recreational athletes of endurance training and physically active men following maximal incremental exercise testing. (Manuscript section: lines 119 to 121).

### ****METHODS****

### ****Study design:**** Observational analytical cross-sectional study with a convenience sampling procedure. (Manuscript section: line 125).

### Setting: We included 53 healthy men (15 control, 15 functional fitness training, and 23 endurance). The collection was carried out in a laboratory room between 08:00 and 10:00 am, room temperature (21 - 24°C) and relative humidity of 50 to 60%. (Manuscript section: lines 123 to 163).

**Participants:** Healthy adult males aged 20 to 40 years. Those who smoked, used medications, and/or had a non-sinus rhythm observed by the electrocardiogram were excluded. (Manuscript section: lines 127 to 131).

**Variables:** Dependent variables were heart rate, SD1, SD2 and HR/LH during the recovery phase. Independent variables: the sport practiced by the volunteers. Confounding variables: age, BMI, TT, R, and HR/LF. (Manuscript section: lines 135 to 231).

**Bias:** Heart rate, heart rate variability indices and ergospirometric variables were recorded by validated and calibrated instruments and subsequently analyzed by specific software. (Manuscript section: lines 178 to 231).

**Study Size:** The sample size calculation ensured a statistical power of 80% and a large effect size of 0.50. (Manuscript section: lines 255 to 258).

**Quantitative variables:** Not applicable.

**Statistical methods:** GLM was used for comparisons between groups and Spearman's correlation was used to evaluate the influence of confounding variables on dependent variables. (Manuscript section: lines 238 to 254).

### ****RESULTS****

### ****Participants:** 53 healthy men completed the study**.**** (Manuscript section: line 126).

### Descriptive data: The data were described in table 1 and throughout the text of the results section. (Manuscript section: lines 262 to 275).

### ****Outcome data:** The data were represented by figures 1, 2 and 3 and described throughout the results section.** (Manuscript section: lines 281 to 297).

**Main results:** Heart rate recovery and sympathetic tone withdrawal were significantly lower in the functional training group, indicating lower autonomic efficiency compared to endurance athletes. (Manuscript section: lines 281 to 297).

**Other analyses:** Not applied.

### ****DISCUSSION****

**Key results:** The study demonstrated that endurance athletes have a faster and more efficient cardiac and autonomic recovery than functional training athletes. (Manuscript section: lines 304 to 316).

**Limitations:** The cross-sectional design, a non-random sample, and the lack of individual control of training sessions associated with checking possible signs of overtraining. (Manuscript section: lines 412 to 414).

**Interpretation:** Considering our findings and the popularity of FFT, it is essential to consider that many potential FFT practitioners may have subclinical and asymptomatic cardiovascular conditions that must be taken into account when the exercise regime may be associated with delayed recovery. (Manuscript section: lines 420 to 423).

**Generalisability:** The results cannot be generalized to men and women of different ages who engage in fitness-functional training at an athletic level. (Manuscript section: lines 428 to 429).

### ****OTHER INFORMATION****

### ****Funding:** Not Applied due to the journal's manuscript guidelines.**
