# Supplementary material for: Delayed heart rate recovery and its variability in fitness functional training compared to endurance athletes: a cross-sectional analysis
Source: PeerJ. 2026 Jan 5;14:e20335. doi: 10.7717/peerj.20335 (PMC12782030; doi:10.7717/peerj.20335)
Supplement: Supplemental Information 3 — Values for the control group (CG), endurance group (EG), and fitness functional group (FFG) are presented as medians (25th, 75th percentiles). Group comparisons were performed using a Generalized Linear Model (GLM) adjusted for age, BMI, total exercise time (TT), respiratory exchange ratio (R), and HR/LF at peak exercise. β (95% CI): difference between estimated means with 95% confidence interval. p, p-value for least significant difference post hoc test; *, p-value statistically different (p ≤ 0.05) for least significant difference post hoc test; HRR: absolute heart rate recovery; % HRR, relative heart rate recovery; SD1, instantaneous index of R-R series variability; SD2, measures the length from the Poincaré plot; HR/LF, heart rate/low-frequency ratio. [file peerj-14-20335-s003.docx]

| **Chronotropic response** |  | **CG (n=15)** |  | **EG (n=23)** |  | **FFG (n=15)** |  | **β (95%CI)** |  | **p*** |
| --- | --- | --- | --- | --- | --- | --- | --- | --- | --- | --- |
|  |  |  |  |  |  |  |  |  |  |  |
| HRR 1^st^ minute (bpm) |  | 33 (23, 35.50) |  | 31 (26.5, 34.5) |  | 22 (17.5, 28.5) |  | 1.00 (-7.62, 10.63) |  | CG vs. EG = 0.81 |
|  |  |  |  |  |  |  |  | 10.47 (0.42, 21.19) |  | CG vs. FFG = 0.03* |
|  |  |  |  |  |  |  |  | 9.47 (2.00, 16.47) |  | EG vs. FFG = 0.01* |
|  |  |  |  |  |  |  |  |  |  |  |
| HRR 2^nd^ minute (bpm) |  | 51 (46.5, 61.5) |  | 59 (54.5, 64.5) |  | 43 (38.5, 51.5) |  | -6.87 (-15.49, 1.75) |  | CG vs. EG = 0.12 |
|  |  |  |  |  |  |  |  | 6.37 (-4.55, 17.29) |  | CG vs. FFG = 0.25 |
|  |  |  |  |  |  |  |  | 13.24 (4.41, 22.07) |  | EG vs. FFG = 0.01* |
|  |  |  |  |  |  |  |  |  |  |  |
| HRR 3^rd^ minute (bpm) |  | 64 (61, 72) |  | 72 (66.5, 75) |  | 56 (50.5, 63.5) |  | -7.45 (-15.52, 0.62) |  | CG vs. EG = 0.07 |
|  |  |  |  |  |  |  |  | 6.30 (-3.94, 16.53) |  | CG vs. FFG = 0.23 |
|  |  |  |  |  |  |  |  | 13.74 (5.47, 22.02) |  | EG vs. FFG = 0.01* |
|  |  |  |  |  |  |  |  |  |  |  |
| HRR 4^th^ minute (bpm) |  | 69 (67, 76.5) |  | 74 (70.5, 79) |  | 63 (57.5, 68.5) |  | -6.33 (-12.89, 0.23) |  | CG vs. EG = 0.06 |
|  |  |  |  |  |  |  |  | 4.08 (-4.23, 12.40) |  | CG vs. FFG = 0.34 |
|  |  |  |  |  |  |  |  | 10.42 (3.70, 17.14) |  | EG vs. FFG = 0.00* |
|  |  |  |  |  |  |  |  |  |  |  |
| HRR 5^th^ minute (bpm) |  | 75 (66, 81.5) |  | 77 (73, 81.5) |  | 65 (62, 73) |  | -5.46 (-12.68, 1.77) |  | CG vs. EG = 0.14 |
|  |  |  |  |  |  |  |  | 3.38 (-5.78, 12.53) |  | CG vs. FFG = 0.47 |
|  |  |  |  |  |  |  |  | 8.83 (1.43, 16.24) |  | EG vs. FFG = 0.02* |
|  |  |  |  |  |  |  |  |  |  |  |
| % HRR 1^st^ minute (bpm) |  | 17.19 (12.6, 19.4) |  | 16.85 (14.62, 19.32) |  | 11.89 (9.54, 15.98) |  | 0.84 (-3.98, 6.20) |  | CG vs. EG = 0.71 |
|  |  |  |  |  |  |  |  | 5.87 (0.20, 11.87) |  | CG vs. FFG = 0.03* |
|  |  |  |  |  |  |  |  | 5.03 (0.85, 8.95) |  | EG vs. FFG = 0.01* |
|  |  |  |  |  |  |  |  |  |  |  |
| % HRR 2^nd^ minute (bpm) |  | 27.87 (25.62, 33.51) |  | 32.96 (29.16, 35.73) |  | 23.24 (20.44, 30.21) |  | -3.55 (-8.30, 1.19) |  | CG vs. EG = 0.14 |
|  |  |  |  |  |  |  |  | 3.27 (-2.74, 9.29) |  | CG vs. FFG = 0.29 |
|  |  |  |  |  |  |  |  | 6.83 (1.96, 11.69) |  | EG vs. FFG = 0.01* |
|  |  |  |  |  |  |  |  |  |  |  |
| % HRR 3^rd^ minute (bpm) |  | 35.56 (32.53, 39.25) |  | 39.66 (36.99, 41.71) |  | 30.27 (26.93, 36.32) |  | -3.72 (-8.29, 0.85) |  | CG vs. EG = 0.11 |
|  |  |  |  |  |  |  |  | 3.26 (-2.54, 9.05) |  | CG vs. FFG = 0.27 |
|  |  |  |  |  |  |  |  | 6.98 (2.29, 11.66) |  | EG vs. FFG = 0.00* |
|  |  |  |  |  |  |  |  |  |  |  |
| % HRR 4^th^ minute (bpm) |  | 38.33 (35.44, 41.54) |  | 41.52 (39.72, 42.86) |  | 34.05 (31.15, 38.81) |  | -3.03 (-6.76, 0.69) |  | CG vs. EG = 0.11 |
|  |  |  |  |  |  |  |  | 2.07 (-2.65, 6.79) |  | CG vs. FFG = 0.39 |
|  |  |  |  |  |  |  |  | 5.10 (1.28, 8.92) |  | EG vs. FFG = 0.01* |
|  |  |  |  |  |  |  |  |  |  |  |
| % HRR 5^th^ minute (bpm) |  | 39.68 (35.19, 44.93) |  | 42.46 (40.82, 44.93) |  | 35.2 (33.9, 40.91) |  | -2.53 (-6.63, 1.58) |  | CG vs. EG = 0.23 |
|  |  |  |  |  |  |  |  | 1.70 (-3.51, 6.90) |  | CG vs. FFG = 0.52 |
|  |  |  |  |  |  |  |  | 4.22 (0.01, 8.43) |  | EG vs. FFG = 0.05* |
|  |  |  |  |  |  |  |  |  |  |  |
| **Autonomic response** |  | **CG (n=15)** |  | **EG (n=23)** |  | **FFG (n=15)** |  | **β (95%CI)** |  | **P** |
|  |  |  |  |  |  |  |  |  |  |  |
|  |  |  |  |  |  |  |  |  |  |  |
| SD1 1^st^ minute (ms) |  | 2.5 (1.8, 2.95) |  | 3.2 (2.65, 4) |  | 3.2 (2.6, 3.5) |  | -0.84 (-1.53, -0.12) |  | CG vs. EG = 0.02* |
|  |  |  |  |  |  |  |  | -0.78 (-1.65, 0.12) |  | CG vs. FFG = 0.10 |
|  |  |  |  |  |  |  |  | 0.06 (-0.81, 0.89) |  | EG vs. FFG = 0.88 |
|  |  |  |  |  |  |  |  |  |  |  |
| SD1 2^nd^ minute (ms) |  | 2.5 (2.1, 3.1) |  | 3.6 (2.6, 4.6) |  | 3.4 (2.4, 4.6) |  | -0.61 (-1.81, 0.70) |  | CG vs. EG = 0.32 |
|  |  |  |  |  |  |  |  | 0.05 (-1.38, 1.56) |  | CG vs. FFG = 0.94 |
|  |  |  |  |  |  |  |  | 0.66 (-0.73, 1.94) |  | EG vs. FFG = 0.32 |
|  |  |  |  |  |  |  |  |  |  |  |
| SD1 3^rd^ minute (ms) |  | 2.8 (2.25, 3.2) |  | 3.8 (3.2, 4.4) |  | 3.5 (2.55, 4.4) |  | -0.80 (-2.37, 0.92) |  | CG vs. EG = 0.28 |
|  |  |  |  |  |  |  |  | 0.21 (-1.58, 2.14) |  | CG vs. FFG = 0.81 |
|  |  |  |  |  |  |  |  | 1.02 (-0.69, 2.61) |  | EG vs. FFG = 0.20 |
|  |  |  |  |  |  |  |  |  |  |  |
| SD1 4^th^ minute (ms) |  | 2.8 (2.4, 4.3) |  | 4.3 (3.35, 5) |  | 3.8 (2.9, 4.95) |  | -0.58 (-2.26, 1.21) |  | CG vs. EG = 0.46 |
|  |  |  |  |  |  |  |  | -0.28 (-2.23, 1.75) |  | CG vs. FFG = 0.77 |
|  |  |  |  |  |  |  |  | 0.30 (-1.52, 1.99) |  | EG vs. FFG = 0.73 |
|  |  |  |  |  |  |  |  |  |  |  |
| SD1 5^th^ minute (ms) |  | 2.8 (2.6, 4.6) |  | 4.3 (3.45, 5.1) |  | 4.1 (2.9, 5.25) |  | -0.65 (-2.35, 1.17) |  | CG vs. EG = 0.42 |
|  |  |  |  |  |  |  |  | -0.49 (-2.49, 1.59) |  | CG vs. FFG = 0.61 |
|  |  |  |  |  |  |  |  | 0.16 (-1.74, 1.92) |  | EG vs. FFG = 0.86 |
|  |  |  |  |  |  |  |  |  |  |  |
| SD2 1^st^ minute (ms) |  | 2.7 (2.3, 3.8) |  | 3.1 (2.25, 3.7) |  | 2.4 (2.15, 2.8) |  | -0.08 (-1.32, 1.31) |  | CG vs. EG = 0.90 |
|  |  |  |  |  |  |  |  | 0.84 (-0.50, 2.34) |  | CG vs. FFG = 0.25 |
|  |  |  |  |  |  |  |  | 0.92 (-0.30, 2.05) |  | EG vs. FFG = 0.12 |
|  |  |  |  |  |  |  |  |  |  |  |
| SD2 2^nd^ minute (ms) |  | 3.9 (3.35, 4.95) |  | 4.9 (3.95, 5.8) |  | 3.5 (2.7, 4.75) |  | -0.47 (-3.04, 2.50) |  | CG vs. EG = 0.70 |
|  |  |  |  |  |  |  |  | 2.04 (-0.76, 5.16) |  | CG vs. FFG = 0.13 |
|  |  |  |  |  |  |  |  | 2.51 (0.17, 4.68) |  | EG vs. FFG = 0.02* |
|  |  |  |  |  |  |  |  |  |  |  |
| SD2 3^rd^ minute (ms) |  | 4.7 (3.95, 5.7) |  | 5.8 (4.75, 8.9) |  | 3.9 (3.3, 5.45) |  | -1.15 (-4.68, 2.84) |  | CG vs. EG = 0.48 |
|  |  |  |  |  |  |  |  | 2.05 (-1.78, 6.23) |  | CG vs. FFG = 0.23 |
|  |  |  |  |  |  |  |  | 3.20 (0.28, 6.13) |  | EG vs. FFG = 0.03* |
|  |  |  |  |  |  |  |  |  |  |  |
| SD2 4^th^ minute (ms) |  | 5.4 (4.65, 6.2) |  | 6.3 (5.05, 8.75) |  | 5.4 (4.05, 6.55) |  | -0.98 (-4.59, 3.06) |  | CG vs. EG = 0.56 |
|  |  |  |  |  |  |  |  | 1.67 (-2.48, 5.97) |  | CG vs. FFG = 0.36 |
|  |  |  |  |  |  |  |  | 2.65 (-0.99, 5.97) |  | EG vs. FFG = 0.10 |
|  |  |  |  |  |  |  |  |  |  |  |
| SD2 5^th^ minute (ms) |  | 5.5 (5, 7.35) |  | 6.8 (5.35, 8.75) |  | 6.3 (4.4, 7.5) |  | -0.90 (-4.36, 3.02) |  | CG vs. EG = 0.58 |
|  |  |  |  |  |  |  |  | 1.01 (-3.14, 5.40) |  | CG vs. FFG = 0.58 |
|  |  |  |  |  |  |  |  | 1.91 (-1.74, 5.26) |  | EG vs. FFG = 0.24 |
|  |  |  |  |  |  |  |  |  |  |  |
| HR/LF 1^st^ minute (ms) |  | 202.34 (37.95, 387.57) |  | 271.98 (123.53, 595.04) |  | 641.54 (321.85, 1184.63) |  | -432.38 (-689.13, -165.73) |  | CG vs. EG = 0.00* |
|  |  |  |  |  |  |  |  | -1104.49 (-1871.29, -337.69) |  | CG vs. FFG = 0.00* |
|  |  |  |  |  |  |  |  | -672.11 (-1491.85, 147.63) |  | EG vs. FFG = 0.10 |
|  |  |  |  |  |  |  |  |  |  |  |
| HR/LF 2^nd^ minute (ms) |  | 52.56 (25.32, 65.67) |  | 24.39 (12.3, 63.98) |  | 167.03 (37.2, 355.45) |  | 10.62 (-42.22, 63.46) |  | CG vs. EG = 0.68 |
|  |  |  |  |  |  |  |  | -194.69 (-352.01, -37.37) |  | CG vs. FFG = 0.01* |
|  |  |  |  |  |  |  |  | -205.31 (-354.45, -56.16) |  | EG vs. FFG = 0.00* |
|  |  |  |  |  |  |  |  |  |  |  |
| HR/LF 3^rd^ minute (ms) |  | 17.04 (10.17, 41.95) |  | 8.4 (4.65, 24.65) |  | 54.46 (20.36, 107.82) |  | 2.82 (-24.36, 30) |  | CG vs. EG = 0.83 |
|  |  |  |  |  |  |  |  | -60.48 (-122.25, 1.29) |  | CG vs. FFG = 0.05* |
|  |  |  |  |  |  |  |  | -63.30 (-120.09, -6.51) |  | EG vs. FFG = 0.03* |
|  |  |  |  |  |  |  |  |  |  |  |
| HR/LF 4^th^ minute (ms) |  | 11.51 (6.75, 29.47) |  | 7.21 (2.78, 14.01) |  | 32.18 (17.65, 50.84) |  | 5.37 (-11.81, 28.60) |  | CG vs. EG = 0.57 |
|  |  |  |  |  |  |  |  | -23.96 (-62.58, 8.88) |  | CG vs. FFG = 0.17 |
|  |  |  |  |  |  |  |  | -29.33 (-66.79, -2.95) |  | EG vs. FFG = 0.05* |
|  |  |  |  |  |  |  |  |  |  |  |
| HR/LF 5^th^ minute (ms) |  | 8.69 (5.06, 17.87) |  | 5.33 (2.65, 14.25) |  | 18.11 (10.26, 29.82) |  | 2.78 (-8.15, 17.61) |  | CG vs. EG = 0.66 |
|  |  |  |  |  |  |  |  | -11.69 (-32.75, 7.93) |  | CG vs. FFG = 0.27 |
|  |  |  |  |  |  |  |  | -14.47 (-34.36, 1.48) |  | EG vs. FFG = 0.10 |
